# Knee and Hip Joint Kinematics Predict Quadriceps and Hamstrings Neuromuscular Activation Patterns in Drop Jump Landings

**DOI:** 10.1371/journal.pone.0153737

**Published:** 2016-04-21

**Authors:** Bart Malfait, Bart Dingenen, Annemie Smeets, Filip Staes, Todd Pataky, Mark A. Robinson, Jos Vanrenterghem, Sabine Verschueren

**Affiliations:** 1 Musculoskeletal Rehabilitation Research Group, Department of Rehabilitation Sciences and Physiotherapy, Faculty of Kinesiology and Rehabilitation Sciences, KU Leuven, Belgium; 2 Research Institute for Sport and Exercise Sciences, Faculty of Science, Liverpool John Moores University, Liverpool, United Kingdom; 3 Department of Bioengineering, Shinshu University, Ueda, Japan; Universite de Nantes, FRANCE

## Abstract

**Purpose:**

The purpose was to assess if variation in sagittal plane landing kinematics is associated with variation in neuromuscular activation patterns of the quadriceps-hamstrings muscle groups during drop vertical jumps (DVJ).

**Methods:**

Fifty female athletes performed three DVJ. The relationship between peak knee and hip flexion angles and the amplitude of four EMG vectors was investigated with trajectory-level canonical correlation analyses over the entire time period of the landing phase. EMG vectors consisted of the {vastus medialis(VM),vastus lateralis(VL)}, {vastus medialis(VM),hamstring medialis(HM)}, {hamstring medialis(HM),hamstring lateralis(HL)} and the {vastus lateralis(VL),hamstring lateralis(HL)}. To estimate the contribution of each individual muscle, linear regressions were also conducted using one-dimensional statistical parametric mapping.

**Results:**

The peak knee flexion angle was significantly positively associated with the amplitudes of the {VM,HM} and {HM,HL} during the preparatory and initial contact phase and with the {VL,HL} vector during the peak loading phase (p<0.05). Small peak knee flexion angles were significantly associated with higher HM amplitudes during the preparatory and initial contact phase (p<0.001). The amplitudes of the {VM,VL} and {VL,HL} were significantly positively associated with the peak hip flexion angle during the peak loading phase (p<0.05). Small peak hip flexion angles were significantly associated with higher VL amplitudes during the peak loading phase (p = 0.001). Higher external knee abduction and flexion moments were found in participants landing with less flexed knee and hip joints (p<0.001).

**Conclusion:**

This study demonstrated clear associations between neuromuscular activation patterns and landing kinematics in the sagittal plane during specific parts of the landing. These findings have indicated that an erect landing pattern, characterized by less hip and knee flexion, was significantly associated with an increased medial and posterior neuromuscular activation (dominant hamstrings medialis activity) during the preparatory and initial contact phase and an increased lateral neuromuscular activation (dominant vastus lateralis activity) during the peak loading phase.

## Introduction

Anterior cruciate ligament (ACL) injuries are very common during dynamic sports activities in the active population (16–39 years) accounting for approximately 26% of all internal knee injuries [1]. ACL injuries may have important short and long-term physical, psychological and professional consequences [2] for the injured athletes resulting in a substantial, long withdrawal from sports and high economic costs for society [3]. Therefore, screening programs have been developed in an attempt to determine ACL injury risk. Recent literature has extensively investigated the ACL injury mechanism [4]. Non-contact ACL injuries represent 70% of all ACL injuries [5]. They commonly occur during landing activities, more specifically in the deceleration phase immediately after initial ground contact [5]. A prospective study by Hewett et al. [6] has shown that high knee abduction moments during landing of drop vertical jumps (DVJ) increase ACL injury risk. Additionally, a more erect landing pattern, characterized by more extended knee, hip and trunk positions, increases the vertical ground reaction force [6], external knee flexion moment [7], external knee abduction moment [8,9] and the anterior tibial shear force [10], all of which might be risk factors for ACL injury [11]. Blackburn et al. [12] have shown that peak knee and hip flexion angles during landing, two easy to measure parameters, influence the kinetics at the hip and knee joints resulting in a higher injury risk. However, this study was conducted while participants were not allowed to perform the DVJ naturally in their preferred way; a specific trunk flexed pattern was instructed [12]. Because of the coupling of the knee and hip joints in the closed-kinetic chain, active trunk flexion during landing produces concomitant increases in knee and hip flexion angles compared to a more erect trunk posture [13]. If the knee moves into more flexion during the loading phase of a landing, the anterior tibial shear force decreases [14], and thus injury risk might be reduced. Furthermore, intervention studies have shown that a combination of strengthening exercises, proximal control exercises and exercises that improve the landing pattern (such as a more flexed landing pattern) can reduce ACL injury risk [15,16].

Many biomechanical risk factors for ACL injury have been proposed, however few studies have examined muscular activation patterns that might be related to ACL injury risk. Besides the external forces acting on the knee joint during dynamic activities, the quadriceps and hamstrings muscle groups have the potential to either load or unload the knee ligaments based on their coordinated activation. Several cadaveric [17] and in-vivo studies [14] have shown that quadriceps contraction induces tension and strain to the ACL. Furthermore, previous studies suggested that the hamstrings muscles might counteract the anterior shear force that strains the ACL by creating a posteriorly orientated force [18]. Quadriceps and hamstrings co-contractions can therefore be effective in reducing these excessive in-situ forces in the ACL, and this particularly when the knee is more flexed (>15° of knee flexion) [17]. In addition to the anterior and posterior (un)loading support of the quadriceps and hamstrings complex, Lloyd et al. [19] have shown that the quadriceps and hamstrings muscle groups also have adduction and abduction moment arms potentially influencing the knee adduction/abduction loading.

A prospective study by Zebis et al. [20] showed that a large difference in muscular activity (amplitude) between the vastus lateralis (VL) and the musculus semitendinosus (ST) during the preparatory phase (10 ms before initial contact) of a side cutting manoeuver might have a predictive value for ACL injury risk determination [20]. As most ACL injuries occur within 40 ms after initial contact [4], and literature has shown that this time period is too short for mechanosensory feedback to control the knee joint during functional sports activities [21], the neuromuscular coordination during the preparatory phase before initial contact might be crucial for injury prevention.

Despite literature that suggested high risk neuromuscular activation patterns during DVJ, the relation between sagittal plane landing kinematics and muscular activation patterns of the quadriceps and hamstrings muscle groups is still not well examined. Recent work of Blackburn et al. [12] showed that trunk flexion, resulting in a less erect landing posture, reduced the vertical ground reaction forces and quadriceps activity during landing. However, because no electromyographic (EMG) measurements of the hamstrings muscles were included in this study, the interaction between quadriceps and hamstrings activation patterns and the lower limb kinematics remains unclear. As previously mentioned, the participants were not allowed to perform the drop landing in their preferred way. Walsh et al. [22] investigated the relationship between muscle activation of gluteus maximus, quadriceps, hamstrings, gastrocnemius and the knee flexion angle during jump landings. They showed that greater mean vastus medialis and gluteus maximus activity during pre-activation was correlated with smaller knee flexion angles (knee extension) at initial contact. Furthermore, preparatory quadriceps/hamstrings co-activation ratio was also negatively correlated with knee flexion angle at initial contact [22].

In the afore mentioned studies [12, 22], EMG data were reduced to a discrete value (e.g. mean or peak value). However, summarizing these complex time-varying multi-dimensional signals to one discrete value that represent the neuromuscular activation of the entire DVJ landing of does not offer an optimal solution [23,24], as neuromuscular activation is constantly adapted to environmental changes through feedforward and feedback mechanisms [25]. In the present study we therefore use a novel statistical method (Statistical Parametric Mapping) to analyze the EMG activation during the entire DVJ landing without reducing the data or selecting a priori time frames in which we expect an association.

To date, only the relationship between individual muscle activations and landing kinematics has been investigated. However, as agonistic and antagonistic muscle pairs constantly interact, significant associations between neuromuscular activation and knee/hip joint flexion angles could be missed if only individual muscle activation is assessed. Therefore, we will use Statistical Parametric Mapping (SPM) in the present study, a method that accounts for inter-muscle covariance by creating anatomically relevant muscle groupings.

We hypothesized that the variation in sagittal plane knee and hip landing kinematics is associated with the EMG activity patterns of the quadriceps and hamstrings muscle group. Based on the results of Blackburn et al. [12] and Walsh et al. [22], we expected that subjects who show a more erect landing pattern will have increased quadriceps and decreased hamstrings activation compared to subjects who have a more flexed landing pattern.

## Materials and Methods

### Participants

Fifty female athletes (22 soccer, 11 handball and 17 volleyball) consented to participate in this study (age = 21.3 ± 3.4 years; height = 1.72 ± 0.1 m; weight = 66.1 ± 8.5 kg). All participants were member of a Belgian elite level team (first national division) and were injury and pain free. Before participating in this study, all participants provided their written informed consent, which was approved by the local ethics committee. Thirteen participants were aged between 16 and 18 years and signed the informed consent themselves under the written permission of their parents, in which the parents indicated that they fully understood the content of the informed consent and agreed to the signature of their children. As such, no informed written consent was obtained from the next of kin, caretakers, or guardians on behalf of the minors/children enrolled in this study. This study conformed to the principles of the declaration Helsinki (1964), was approved by the local ethics committee and registered with reference number S53369. The local ethical committee approved the consent procedure used in this study. Additionally, all data regarding the participants were anonymized.

### Design

Each test session started with a standardized warm-up, which consisted of two series of eight bipedal squats and eight bipedal jumps [26]. Participants were allowed to familiarize with the tasks by performing three practice repetitions before the start of the tests. Body weight and height were measured before the test session by using respectively a scale (SECA, Hamburg, Germany) and a portable stadiometer (SECA, Hamburg, Germany).

DVJ are commonly used for screening in clinical settings to assess injury risk [6, 27,28]. The protocol used in this study have been previously described elsewhere [26] and so is briefly summarized below. For a DVJ, subjects were instructed to drop off a 0.3 m box with their feet initially positioned 0.2 m apart on the box, and upon landing to immediately perform a maximum vertical jump. Subjects were also instructed to reach upwards with both hands, as if performing a block in volleyball [29]. The task was repeated until 3 valid trials were completed. A trial was excluded if subjects jumped off the box instead of just dropping, if both feet did not land on the force plates, if subjects reached upwards with only one hand, or if subjects clearly lost balance or fell during the test [6]. A one-minute rest period between consecutive trials was permitted to avoid fatigue [26]. Participants wore standardized indoor footwear (KELME INDOOR COPA) and where necessary, long hair was tied up to avoid marker occlusion.

Each participant had 44 spherical reflective markers positioned according to the 6-degrees-of-freedom, eight segment ‘Liverpool John Moores University’ model (LJMU model) including feet, upper and lower legs, pelvis and trunk [26]. Segmental coordinate systems were defined as reported previously [30,31] using separate trials for anatomical calibration [32] and for calculating functional hip joint centres [33] and functional knee joint axes [34]. All modelling and analyses were undertaken in Visual 3D (v.4.83, C-MOTION, Germantown, MD, USA) using geometric volumes to represent segments based on cadaver segmental data. Previous work of our research group showed that the LJMU model was highly reliable during DVJ [25].

### Data collection

A wireless EMG system (AURION, Italy) was used to record the muscle activity of the vastus lateralis (VL), vastus medialis (VM), hamstring lateralis (HL) (i.e. biceps femoris) and hamstring medialis (HM) (i.e. semitendinosus) using surface electrodes which were positioned according to the SENIAM guidelines. All electrode locations were shaved and gently cleaned with 70% isopropyl alcohol to reduce skin impedance. Silver-silver chloride, pre-gelled bipolar surface EMG electrodes (Ambu Blue Sensor, Ballerup, Danmark) were placed over the muscle belly and aligned with the longitudinal axis of the muscle, with a center-to-center distance of 0.02 m. The minimum distance between electrode pairs was set at 0.03 m to reduce the possibility of cross-talk.

DVJ were completed on two individual 0.8 x 0.3 m^2^ AMTI (Watertown, MA, USA) force plates. Force plate and EMG data were sampled at 1000 Hz. Three-dimensional kinematic data were simultaneously (time synchronized) recorded with the force and EMG data in Nexus (VICON, Oxford Metrics, UK) using 6 MX-T20 optoelectronic cameras (VICON, Oxford Metrics, UK) sampling at 100 Hz.

### Data analysis

**Fig 1:**
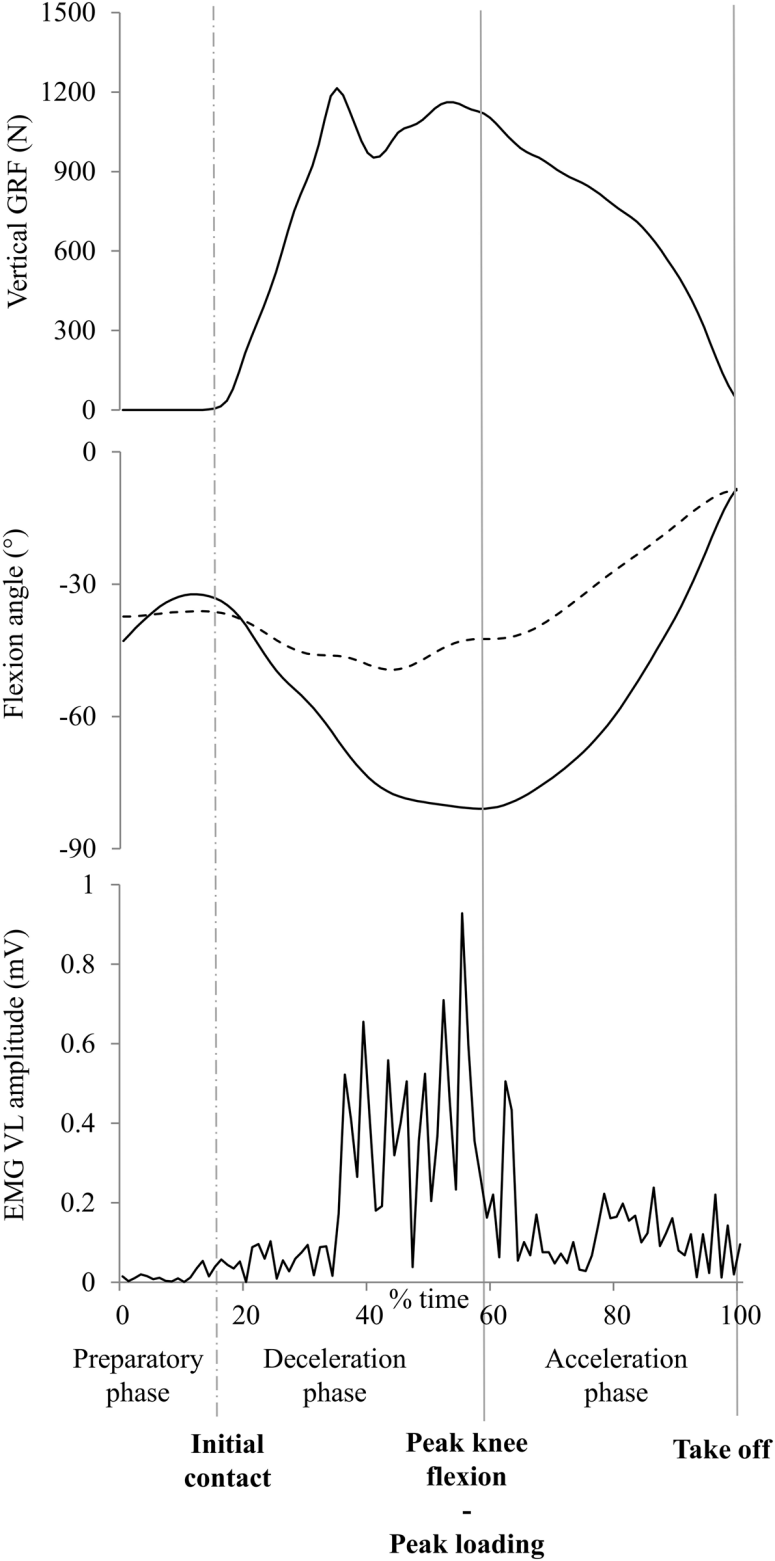
Time periods during DVJ. Data of 1 representative participant to illustrate the different time periods during DVJ from 100ms before initial contact until take off. The graph of the flexion angle represents both the knee and hip flexion angle. The dotted line represents the hip flexion angle.

Only the first landing (first contact) within each DVJ trial was used for analysis [26]. Whole-body kinematics and kinetics were collected and processed in accordance to literature convention, however only the dominant leg was analysed and this was defined as the preferred leg to kick a ball [35]. Marker trajectories and forces were filtered using a 4^th^ order low pass Butterworth filter with a cut-off frequency of 18 Hz [36]. Initial contact and take off events were created when the vertical force crossed a 20 N threshold. All raw EMG signals of the DVJ trials and all raw EMG signals of the maximum voluntary contraction (MVC) trials were high pass filtered at a cut-off frequency of 10 Hz. Subsequently, the signals were rectified and low pass filtered with a 4^th^ order zero-lag Butterworth filter at a cut-off frequency of 6 Hz. The EMG signal amplitudes of the DVJ Trials were normalized to the root mean square amplitude (over a period of 100 ms) of the MVC out of 3 attempts. Kinetic and kinematic data were normalized to 100% of the phase starting at 100ms before initial contact until take off as can be seen in Fig 1.

Peak knee and hip flexion angles were calculated because these discrete values illustrate the amount of knee and hip flexion during the landing phase and are easy to measure in a clinical setting [9]. The external knee flexion and knee abduction moments were calculated using inverse dynamics. External joint moments are described in this study; i.e. an external knee abduction load will tend to abduct the knee (move the distal tibia away from the midline of the participant’s body). The peak joint angles and the peak external moments were calculated during the first contact phase on the force plates, between initial contact and take off (Fig 1). Additionally, negative values for knee and hip joint angles indicated knee and hip joint flexion, and less negative values indicated more knee and hip joint extension.

### Statistical analysis

All participants (n = 50) were divided into quintiles based on their landing pattern. Quintile one consisted of the ten participants who demonstrated the highest peak knee/hip flexion angles. Quintile five consisted of the ten participants who demonstrated the lowest knee/hip flexion angles (more extended knee/hip joint angles). Quintiles 2, 3, and 4 demonstrated values ranging from much flexion towards less flexion, respectively.

Shapiro-Wilk analyses were used to test normality of all kinematic and kinetic data of the different quintiles. Independent t-tests were used to compare the kinematics and kinetics between the upper and lower quintiles. Pearson correlation analysis was used to investigate the relation between peak hip and peak knee flexion joint angles in all participants.

**Fig 2:**
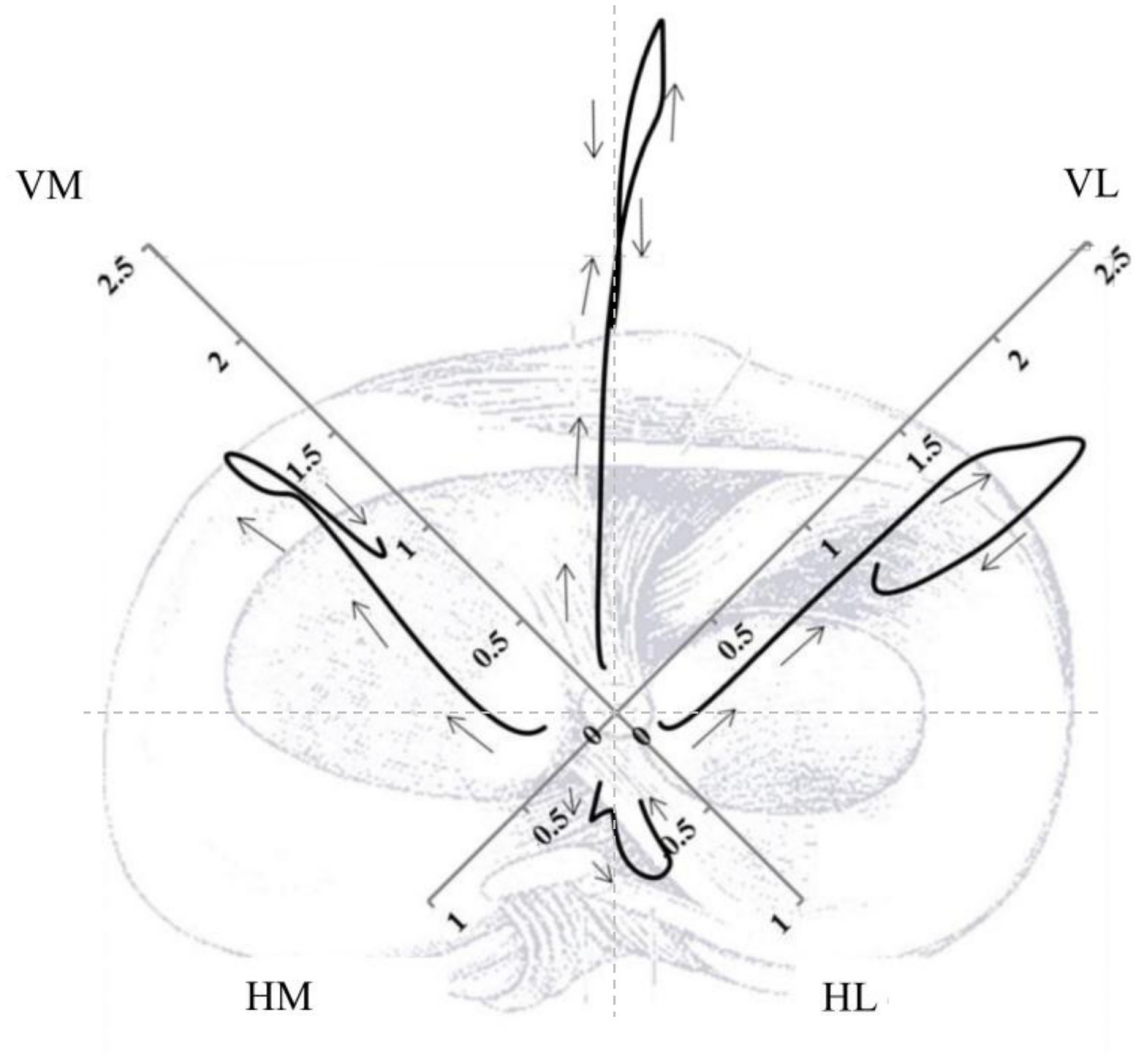
Neuromuscular activation patterns of all participants (n = 50) consisting of four normalized EMG vectors. Each vector represents the activation of muscle pairs throughout the landing phase. The arrows indicate the time component going from 100ms before initial contact until take off.

As we were interested in neuromuscular activation of muscle pairs around the knee rather than the activation of individual muscles, we created four anatomically meaningful EMG vectors: an anterior EMG vector field {VM,VL}, a lateral EMG vector field {VL,HL}, a posterior EMG vector field {HM,HL}, and a medial EMG vector field {HM,VM} as can be seen in Fig 2. This approach accounts for the inter-muscle covariance as well as the time-dependence of multiple EMG signals whilst also controlling Type I and Type II statistical errors resulting in an objective framework for hypothesis evaluation [23].

Furthermore, correlation analyses were used to further assess the relationships between peak knee/hip joint flexion angles and the neuromuscular activation patterns of VL, VM, HL and HM. As we did not want to reduce the EMG data to a discrete value, we used Statistical Parametric Mapping (SPM) a technique that allows us to analyze the entire EMG time series. To avoid multiple EMG signal co-variance bias [23,24] we used the multivariate equivalent of linear regression, canonical correlation analysis (CCA, S1 Appendix). This analysis calculates test statistics e.g. linear regression at each time node, yet elegantly handles the problem of multiple comparisons by modeling the behavior of random time-varying signals [24]. To establish if there was a relationship between the combination of peak knee flexion and peak hip flexion angles and the overall EMG vector, we first analyzed the {peak knee flexion angle, peak hip flexion angle} vector and the {VM,VL,HM,HL} (time) vector field. Subsequently, the correlation between peak knee flexion angle and four anatomically meaningful muscle pair vectors was calculated (anterior EMG vector field {VM,VL}, lateral EMG vector field {VL,HL}, posterior EMG vector field {HM,HL} and a medial EMG vector field {HM,VM}). The same correlation analyses were undertaken for peak hip flexion angles. In total eight analyses were conducted (peak knee flexion angle vs. four EMG vectors, peak hip flexion angle vs. four EMG vectors) and the test statistic measured was the maximum canonical correlation, a single correlation coefficient which varies over time and which can be transformed to the χ^2^ statistic.

Statistical inference was conducted using Random Field Theory [37]. This uses the smoothness of the EMG residual trajectories to determine the critical threshold that Alpha % (5% in this study) of identically smooth random trajectories would exceed. If the test statistic trajectory exceeded the critical threshold, there was a significant linear relationship between the predictor variable and the EMG vector field. Detailed examples, theoretical background and interpretations of vector field and SPM statistics are outlined in more detail elsewhere [23,24]. To estimate the contribution of each individual muscle to the maximum canonical correlation, post-hoc linear regressions were conducted using one-dimensional statistical parametric mapping (1-D SPM). Statistic calculation and statistical inference were similar to those as described above [23,28,38]. All statistical analyses were conducted in Python (v.2.7.2; Enthought Python Distribution, Austin, TX).

## Results

**Fig 3:**
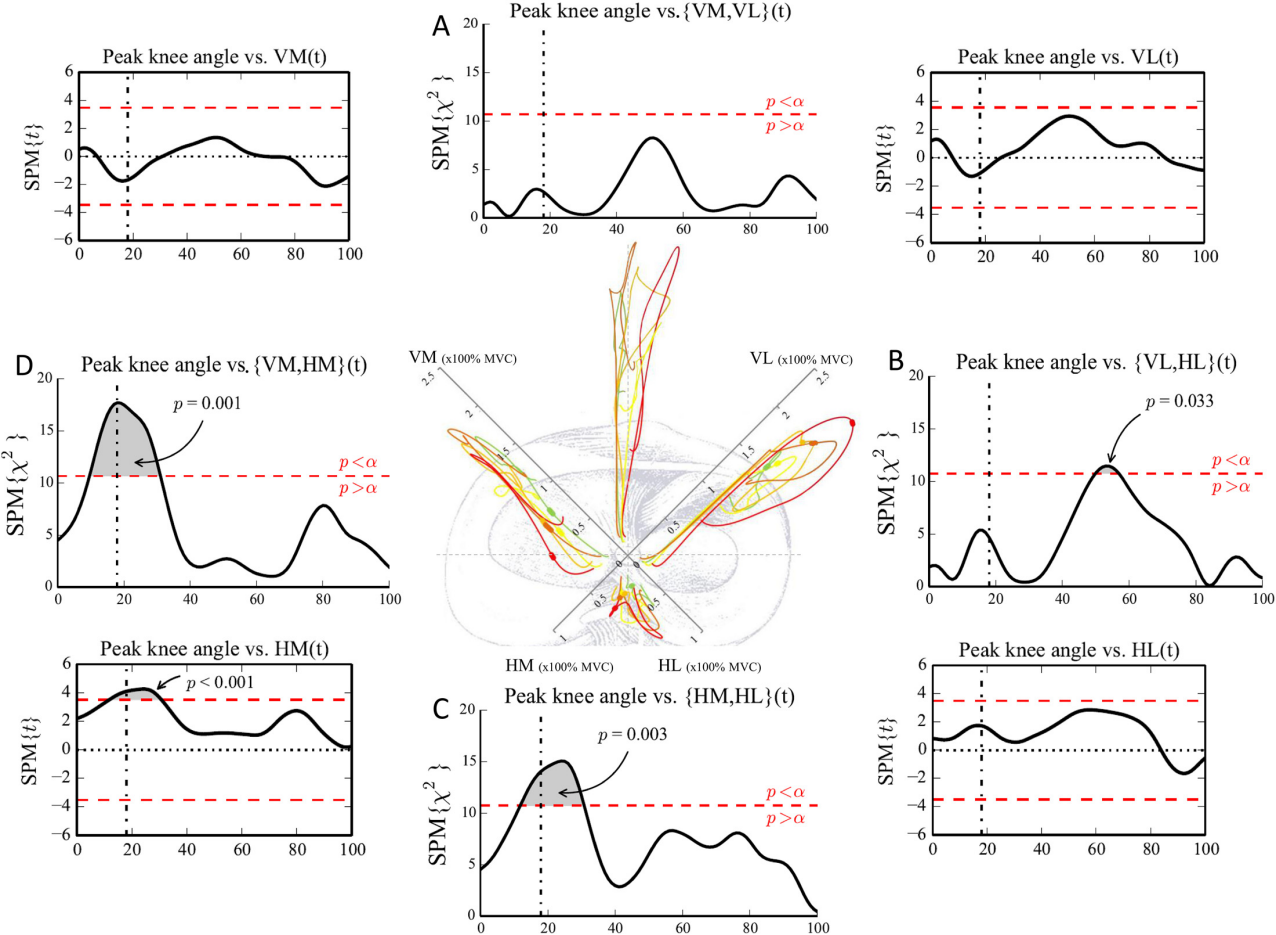
Neuromuscular activation patterns of muscle pairs and the relation with the peak knee joint angle. (Central figure) Differences in activation patterns between the different quintiles are visualized. The participants were divided in quintiles based on the peak knee flexion angles. green (1): largest knee flexion angle; yellow (2); light orange (3); orange (4); red (5): smallest knee flexion angle. The bold part of the quintiles shows the time point where the relation between the EMG vector and the peak knee flexion angle is the most significant. (A,B,C,D) CCA show the association between peak knee joint flexion angle and the anterior {VM,VL}, lateral {VL,HL}, posterior {HM,HL} and medial {VM,HM} EMG vector. The vertical dashed-dotted line represents the initial contact event. The horizontal dashed line represents the critical threshold (p<0.05). (Corner Figures) Linear regression analyses show the association between peak knee flexion angle and the individual amplitudes of VM, VL, HM and HL. The vertical dashed-dotted line represents the initial contact event. The horizontal dashed line represents the critical threshold (p<0.05).

**Fig 4:**
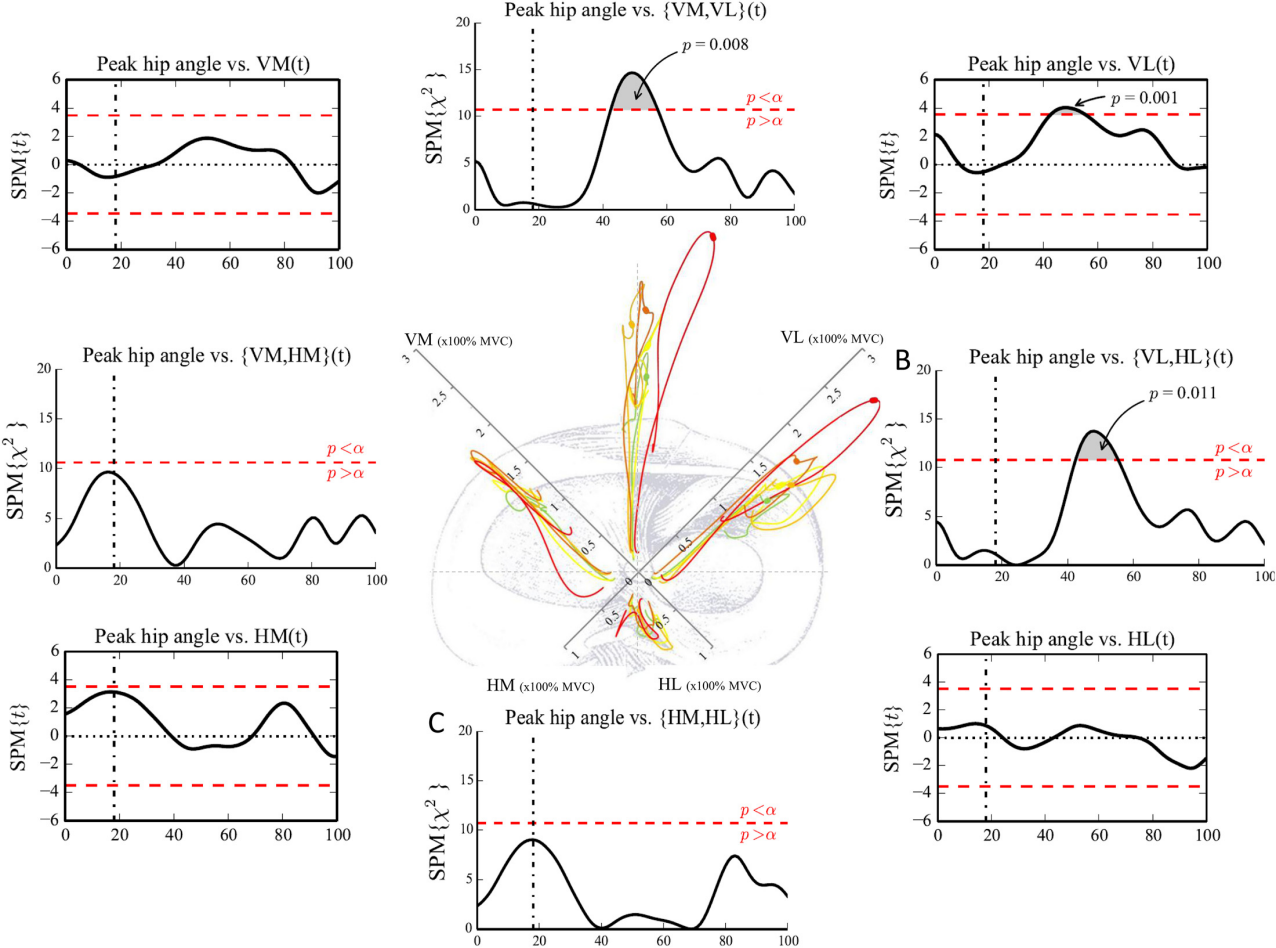
Neuromuscular activation patterns of muscle pairs and the relation with the peak hip joint angle. (Central figure) Differences in activation patterns between the different quintiles are visualized. The participants were divided in quintiles based on the peak hip flexion angles. green (1): largest hip flexion angle; yellow (2); light orange (3); orange (4); red (5): smallest hip flexion angle. The bold part of the quintiles shows the time point where the relation between the EMG vector and the peak hip flexion angle is the most significant. (A,B,C,D) CCA show the association between peak hip joint flexion angle and the anterior {VM,VL}, lateral {VL,HL}, posterior {HM,HL} and medial {VM,HM} EMG vector. The horizontal dashed line represents the critical threshold (p<0.05). (Corner Figures) Linear regression analyses show the association between peak hip flexion angle and the individual amplitudes of VM, VL, HM and HL. The vertical dashed-dotted line represents the initial contact event. The horizontal dashed line represents the critical threshold (p<0.05).

**Fig 5:**
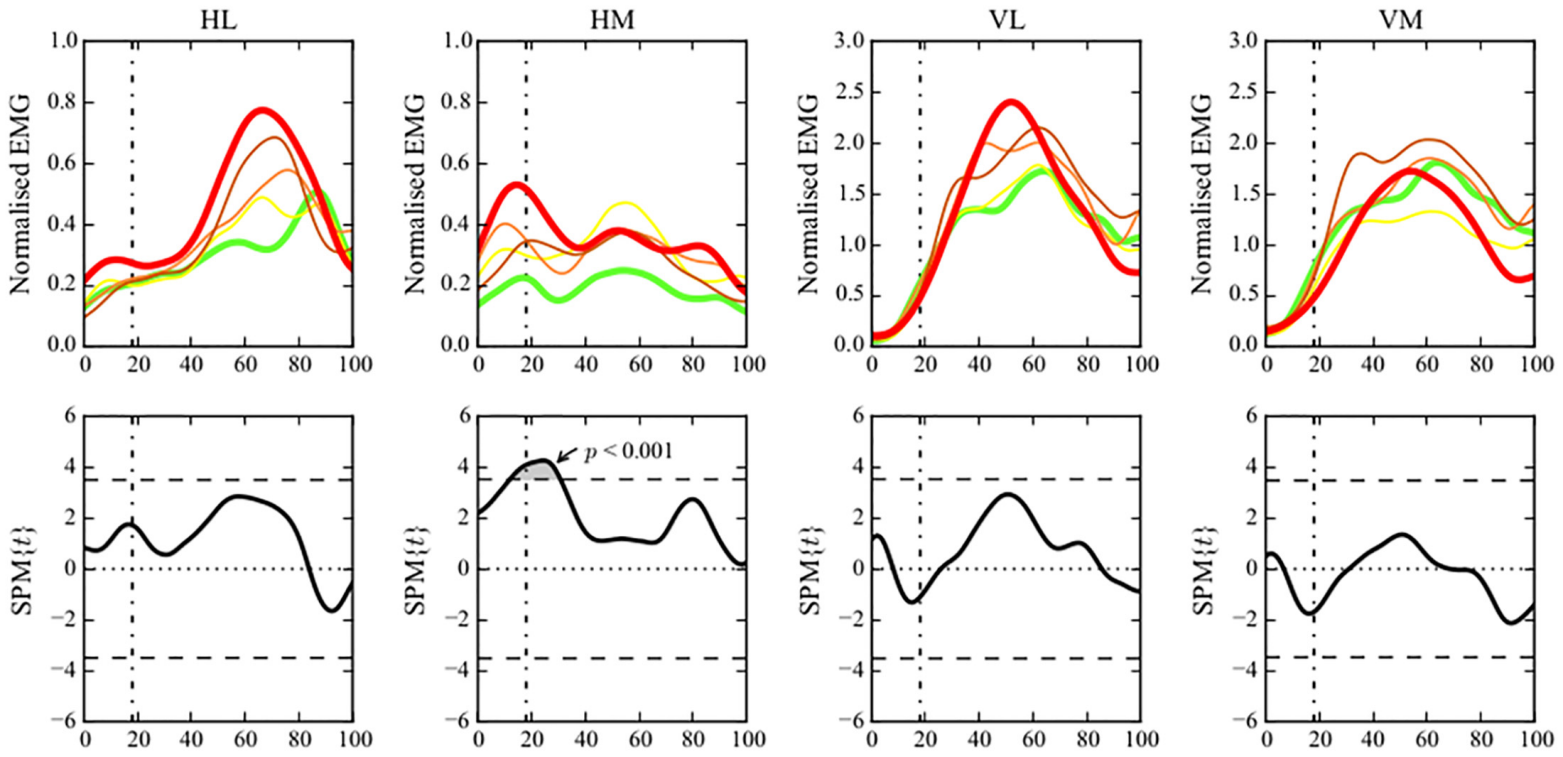
Neuromuscular activation patterns of individual muscles and their relationship with peak knee joint angle. (Upper Row) Visualization of the individual amplitudes of HL, HM, VL and VM for the different quintiles throughout the entire landing phase (from 100ms before initial contact until take off). The participants were divided in quintiles based on the peak knee flexion angles. green (1): largest knee flexion angle; yellow (2); light orange (3); orange (4); red (5): smallest knee flexion angle. (Lower Row) Linear regression analyses show the association between peak knee flexion angle and the individual amplitudes of VM, VL, HM and HL. The vertical dashed-dotted line represents the initial contact event. The horizontal dashed line represents the critical threshold (p<0.05).

**Fig 6:**
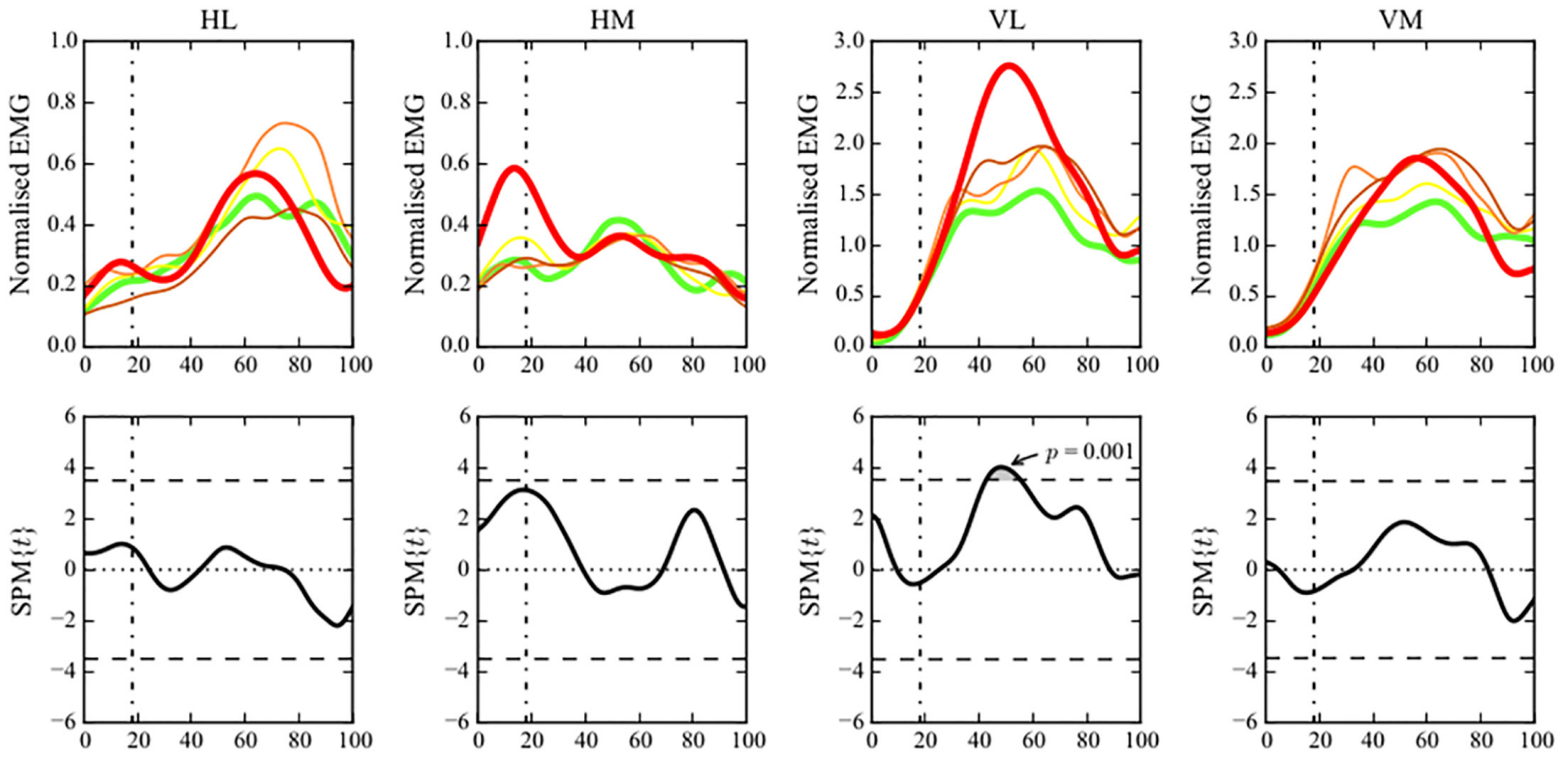
Neuromuscular activation patterns of individual muscles and their relationship with peak hip joint angle. (Upper Row) Visualization of the individual amplitudes of VM, VL, HM, HL for the different quintiles throughout the entire landing phase (from 100ms before initial contact until take off). The participants were divided in quintiles based on the peak hip flexion angles. green (1): largest hip flexion angle; yellow (2); light orange (3); orange (4); red (5): smallest hip flexion angle. (Lower Row) Linear regression analyses show the association between peak hip flexion angle and the individual amplitudes of VM, VL, HM and HL. The vertical dashed-dotted line represents the initial contact event. The horizontal dashed line represents the critical threshold (p<0.05).

Visual observation of the neuromuscular activation patterns of the different quintiles showed differences in both muscle pair activation (Figs 3 and 4) and individual muscle activation (Figs 5 and 6) between the group with a more flexed landing pattern and the group with a more erect landing pattern.

The neuromuscular activation patterns of the different quintiles showed that participants who landed with less peak knee flexion (red line) showed an increased activation of the {VM,HM} vector and the {HM,HL} vector during initial contact and the preparatory phase (Fig 3) mainly due a dominance of HM activity. This indicates that during this specific pre-activity and initial contact phase the HM activity was higher in relation to the activity of the VM (curve of the {VM,HM} vector is more oriented towards the HM axis and thus located under the dashed line in the central figure of Fig 3). Fig 5 (upper row) showed an increased HM activity in the quintile with the lowest peak knee flexion as well. In contrast, after initial contact, an increased activation of the {VM,HM} vector can be observed (Fig 3) mainly due a dominant pattern of the VM. This is seen as the curve is clearly more orientated towards the VM axis (above the dashed line in Fig 3) indicating a higher activity of the VM in relation to the HM activity during the time period following initial contact.

We also found significant associations between peak hip joint angles and neuromuscular activation patterns. Participants who landed with less peak hip flexion (red line) showed a higher activity of the {VM,VL} and the {VL,HL} vector during the peak loading-phase compared to other quintiles. A higher, more dominant activity of the VL was found in the {VM,VL} vector, this indicates a high activity of the VL in relation to the activity of the VM in the upper quintile during the peak loading phase (Fig 4). The increased VL activity in the quintile with more hip extension can be seen in Fig 6 as well.

Tables 1 and 2 show differences in kinematics and kinetics for the participants in the different quintiles. Significantly higher peak external peak knee flexion and abduction moments were found in the groups of athletes who landed with more hip extension (quintile 5) compared with athletes who land with more hip flexion (quintile 1) (Table 2). Subjects who land with more knee extension (quintile 5) have higher external peak knee flexion moments as well (Table 1). An overall significant correlation was found between peak knee and peak hip joint flexion angles (Pearson correlation coefficient, r = 0.65; p<0.001).

**Table 1:** Differences in kinematics and kinetics between the different quintiles based on peak knee flexion angle.

A) Quintiles based on peak knee flexion angle	1 (Flexion)	2	3	4	5 (Extension)	1 vs 5
	Mean ± SD	Mean ± SD	Mean ± SD	Mean ± SD	Mean ± SD	P-value
Peak knee flexion angle (°)	-97.7 ± 4.30	-90.0 ± 2.4	-83.7 ± 1.7	-79.2 ± 1.3	-70.2 ± 5.6	**<0.001**
Peak hip flexion angle (°)	-82.7 ± 11.7	-82.4 ± 9.0	-74.1 ± 13.2	-67.8 ± 9.4	-54.1 ± 14.6	**<0.001**
Relative knee abduction moment (Nm in %BW*Ht)	0.41 ± 0.17	0.28 ± 0.14	0.45 ± 0.12	0.38 ± 0.13	0.56 ± 0.19	0.084
Relative knee flexion moment (Nm in %BW*Ht)	1.22 ± 0.09	1.30 ± 0.16	1.39 ± 0.24	1.38 ± 0.20	1.53 ± 0.31	**0.022**

**Table 2:** Differences in kinematics and kinetics between the different quintiles based on peak hip flexion angle.

B) Quintiles based on peak hip flexion angle	1 (Flexion)	2	3	4	5 (Extension)	1 vs 5
	Mean ± SD	Mean ± SD	Mean ± SD	Mean ± SD	Mean ± SD	P-value
Peak knee flexion angle (°)	-91.2 ± 7.1	-88.0 ± 6.7	-85.1 ± 8.2	-83.8 ± 9.3	-72.7 ± 7.3	**<0.001**
Peak hip flexion angle (°)	-92.9 ± 5.3	-81.0 ± 1.7	-73.9 ± 1.7	-65.9 ± 3.2	-47.4 ± 7.5	**<0.001**
Relative knee abduction moment (Nm in %BW*Ht)	0.37 ± 0.11	0.43 ± 0.17	0.33 ± 0.19	0.38 ± 0.10	0.57 ± 0.19	**0.011**
Relative knee flexion moment (Nm in %BW*Ht)	1.26 ± 0.09	1.32 ± 0.21	1.31 ± 0.22	1.42 ± 0.20	1.52 ± 0.30	**0.028**

BW: body weight;

Ht: height;

SD: standard deviation.

Canonical correlation analysis showed a significant relationship between the {peak knee flexion angle, peak hip flexion angle) vector and the {VM,VL,HM,HL} (time) vector field, we therefore further examined this relationship by considering combinations of EMG components with separate kinematic predictors. Additional CCA showed significant positive associations between peak knee joint flexion angle and the medial, lateral and posterior EMG vector of the quadriceps/hamstrings activation (p<0.05) indicating that muscular activation patterns of {VM,HM}, {VL,HL}, and {HM,HL} are significantly associated with a smaller (i.e. more erect) knee flexion angle during DVJ (Fig 3). More specifically, the lateral activation of {VL,HL} vector showed a significant positive association with the peak knee flexion angle during the peak loading phase (50–60% time) of DVJ (p = 0.033) (Fig 3). The medial activation of {VM,HM} and the posterior activation of {HM,HL} showed significant positive associations with the peak knee joint flexion angle during the preparatory and initial contact phase (10–30% time) (p = 0.001 and p = 0.003, respectively).

Additional 1-D SPM linear regression analyses revealed that the medial hamstring (HM) activity was significantly positively associated with the peak knee joint flexion angle suggesting that less peak knee flexion (i.e. a more extended knee) resulted in more HM activity during the preparatory and initial contact phase (p < 0.001) (Fig 3). In contrast to the significant association between the peak knee flexion angle and the amplitude of the {VL,HL} activation vector during the peak loading phase, no significant associations were found between the peak knee flexion angle and the amplitude of neither the VL nor the HL individually (Fig 3 and S1 Appendix).

As can be seen in Fig 4, peak hip joint flexion angle was significantly (p<0.05) positively associated with the anterior and lateral EMG vector of the quadriceps/hamstrings activation indicating that greater muscular activation patterns of {VM,VL} vector and {VL,HL} vector are significantly associated with a smaller peak hip flexion angle (i.e. more extended hip). The anterior activation of {VM,VL} vector and the lateral activation of {VL,HL} vector showed significant positive associations with the peak hip joint flexion angle specifically during the peak loading phase (approximately 50–60% time) of the DVJ (p = 0.011 and p = 0.008) (Fig 4).

Additional 1-D SPM analyses showed a significant positive association between the VL activity during the peak loading phase of a DVJ and the peak hip joint flexion angle suggesting that athletes who perform DVJ with less peak hip joint flexion (i.e. a more extended hip) show a significantly higher VL activity during the peak loading phase (Fig 4) or vice versa.

## Discussion

The purpose of this study was to assess if sagittal plane landing kinematics of the knee and hip joints may predict neuromuscular activation patterns of quadriceps and hamstrings during the performance of DVJ. First, all subjects were divided into 5 quintiles based on their peak knee and hip flexion angles. The differences in neuromuscular activation, landing kinematics and kinetics were compared between the upper (flexed landing pattern) and lower quintile (erect landing pattern). Subsequently, correlation analyses were used to further assess the relationship between neuromuscular activation patterns and peak hip/knee joint flexion angles.

As we made no hypothesis regarding a specific time point or muscle (pair) a priori, we analyzed the entire landing pattern (from 100ms before initial contact until take off) of four anatomically relevant muscle pairs ({VL, VM}, {VL, HL}, {HM, HL}, {VM, HM}). Subsequently, additional post-hoc 1-D SPM linear regression analyses between the individual muscles and knee/hip joint flexion angles were performed to help interpret the contribution of each individual muscle to the relation between muscle pairs and landing kinematics.

The division of our sample into 5 quintiles based on peak knee and hip flexion angles (Figs 3, 4, 5 and 6) revealed distinct visual differences in EMG vector amplitudes between the two extreme quintiles. The upper quintile (i.e. knee/hip extension angle) showed a clearly increased {VM,HM} and {HM,HL} activation during initial contact and the preparatory phase, mainly due to a dominance of the HM. During the peak loading phase, the upper quintile showed an increased {VL, HL} activation, mainly due to a dominance of the VL (Figs 3, 4, 5 and 6). Additionally, significantly higher external knee flexion and abduction moments were found in the upper quintile (Tables 1 and 2). The distinctive neuromuscular activation patterns found in quintile 5 might be a possible strategy combining feedforward and feedback mechanisms trying to control the high external forces acting on the knee joint in this quintile. Previous studies have shown that a balanced quadriceps/hamstrings activation is very crucial in controlling the external knee flexion and knee abduction moments [19,39].

The results of the correlation analyses further clarify the findings of the quintile analyses. A greater lateral EMG vector ({VL,HL}) during the peak loading phase (50–60% time), and greater medial and posterior EMG vectors ({VM,HM} and {HM,HL}) during the preparatory and initial contact phase (10–30% time) were found in participants who landed with less flexed knee joints (Fig 3). Similarly, participants who landed with less flexed hip joints showed greater anterior and lateral EMG vectors ({VM, VL} and {VL,HL}) during the peak loading phase (Fig 4). Linear regression analyses show that athletes landing in a more erect pattern, i.e. more hip and knee extension [37], demonstrated increased HM activity during the preparatory and initial contact phase (Fig 3) and increased VL amplitude during the peak loading phase (Fig 4), respectively.

Interestingly, in contrast to recent literature [22] a decrease in knee flexion angle towards a more extended knee joint was only significantly associated with a higher HM activity during the preparatory and initial contact phase. Previous in vitro [17] and in vivo [14] studies showed that high quadriceps activity might induce an anteriorly orientated pulling force on the tibia and subsequently strain the ACL during an erect landing. The hamstrings muscle group might counteract the strain on the ACL by creating a posteriorly orientated force on the tibia [40]. A possible explanation for our findings might be the fact that hamstrings are more efficient in producing a counteracting force onto the tibia during more flexed knee joint angles due to the length of the moment arm. This suggests that a higher activity is needed to induce the same posteriorly orientated force when the knee is less flexed and the length of the moment arm is less optimal [41]. Hirokawa et al. [42] showed that hamstrings co-contraction was ineffective in the range of 0°-15° of knee flexion and that the posterior displacement component acting on the tibia was more pronounced in the range of 75°-150° of knee flexion.

Despite the fact that we found an association between the knee flexion angle and the {VL,HL} vector during the peak loading phase, no relations were found between the individual muscle activations of neither the VL nor the HL and the knee joint kinematics (Fig 3). This suggests that a less flexed knee joint during landing is related to an increased {VL,HL} activation in general and not specifically to an increased VL and/or HL activity.

Interestingly, there was a time-shift when comparing the associations between peak knee flexion angles and the HM activity versus the associations between peak knee flexion angles and the {VL,HL} activation (increased HM activity during initial contact and the preparatory phase, increased {VL,HL} activity during the peak loading phase) (Fig 3). On the one hand, the greater level of HM activation prior to landing in participants performing DVJ with more knee extension might indicate that these athletes used a feedforward strategy of HM prior to landing to control the higher ground reaction forces and anterior tibial forces possibly induced by the high quadriceps activity during landing. As previous research has shown [4], the time period between initial contact and moment of injury is often shorter than 50 ms and therefore, preparatory muscular activity might be very important to control the external joint loading. Current results showed significantly higher external peak knee flexion and abduction moments in athletes who performed DVJ with more erect knee and hip joints (Tables 1 and 2). Palmieri-Smith et al. [43] found that during the performance of a forward hop, the medial quadriceps to hamstrings co-contraction index accounts for a significant portion of the variance (R^2^ = 0.792) in peak external knee abduction moment in women. Decreased activation of VM and HM results in a diminished ability to resist external abduction loads. Another study [44] showed that contraction of medial muscles (semitendinosus, medial gastrocnemicus and gracilis muscles) is important in providing resistance to abduction loads.

On the other hand, participants who landed with less knee and hip flexion showed a significantly higher {VL,HL} vector during the peak loading phase. During this particular time phase of the landing, the {VM,VL} vector and VL amplitude were increased as well in the quintile that landed with less knee flexion. Previous studies [12,17] demonstrated that high quadriceps activity might strain the ACL especially when the knee joint flexion angles were smaller than 60°. In addition, disproportional VL activation influences the proximal tibia anterior shear force [45], which in turn is an important loading mechanism of the ACL [46]. Furthermore in a study of Myer et al. [47] female athletes showed decreased medial to lateral quadriceps activation compared to male athletes during a functional knee-extension test. This unbalanced quadriceps activation pattern is suggested to contribute to the increased ACL injury risk in women [47]. Future prospective studies have to confirm this hypothesis.

Interestingly where we did find relations with the VL activity, no significant relations were found between the kinematic parameters and the individual VM activity. Recent work of Beaulieu et al. [39] showed that a lateral/medial imbalance of the muscle activity of the vasti might generate knee abduction moments during cutting manoeuvers, owing to their frontal plane moment arm. Lloyd et al. [19] showed that the knee adduction/abduction moment arms of the quadriceps muscle group are larger at more extended knee angles because the individual muscles tend to be more perpendicular to the tibial plateau compared to more flexed knee positions. This suggests that the quadriceps muscle group has a mechanical advantage to induce knee adduction/abduction load towards more extended knee joints which could explain the findings of our study (higher VL activity was associated with a more extended hip joint during landing and concomitant higher external knee flexion and abduction moments).

Previous intervention studies have shown that movement re-education programs can successfully increase knee and hip joint flexion angles [48] and decrease external knee abduction moments during landing of DVJ [49]. Therefore, based on our results and based on previous intervention studies, we suggest that prevention programs should focus on the improvement of landing patterns towards a more flexed landing pattern. Further research is recommended to investigate the effect of these programs on both the biomechanical and neuromuscular levels.

To our knowledge, this study is the first to comprehensively reveal how neuromuscular activation patterns relate to the preferred landing pattern during the performance of DVJ. However the study still has some limitations that need to be taken into account. Firstly, as the human body acts as a linked-segment model, it might be important to implement more muscles and other joints than the knee and hip joint into the analyses. Less optimal movement patterns of the entire kinetic chain -including ankle, knee, hip and trunk- may contribute to ACL injury risk. Previous studies showed an influence of other proximal and distal musculature on the knee joint [40]. Further research should include activation patterns of other relevant muscle groups such as the gastrocnemius, soleus and glutei for example which could influence the knee joint kinematics and kinetics, to enlarge our knowledge about the link between basic kinematics and neuromuscular activation patterns. Secondly, DVJ were used in this study because of the good reliability of this dynamic screening task which is commonly used in the literature focusing on injury risk assessment and prevention [26]. However, the results concerning the activation patterns are task specific and should likely not be generalized towards other screening tasks without caution. Finally, it still needs to be determined prospectively if an increased HM activity during the initial and preparatory phase and an increased VL activity at peak loading can predict an increased ACL injury risk.

## Conclusions

The current study has demonstrated clear associations during specific time periods between neuromuscular activation patterns and landing kinematics in the sagittal plane. The present findings have indicated that an erect landing pattern, characterized by less hip and knee flexion, was significantly associated with an increased activation of medial {VM, HM} and posterior {HM, HL} muscle pairs during the preparatory and initial contact phase and a more dominant and increased activation of anterior {VM, VL} and lateral {VL, HL} muscle pairs during the peak loading phase, respectively. Post-hoc analysis showed that an increased HM activation was mainly responsible for the increased medial and posterior activation during the preparatory and initial contact phase, and an increased VL activation was responsible for the increased anterior and lateral activation during peak loading. This suggests that participants landing in an erect pattern perform dynamic tasks with different neuromuscular activation patterns of the quadriceps/hamstrings complex. Future prospective studies should investigate if specific neuromuscular landing patterns are related to a higher ACL injury risk.

## Supporting Information

S1 Appendix Canonical Correlation Analysis (CCA) versus linear regression. (PDF)
