# Supplementary material for: Knee and Hip Joint Kinematics Predict Quadriceps and Hamstrings Neuromuscular Activation Patterns in Drop Jump Landings
Source: PLoS One. 2016 Apr 21;11(4):e0153737. doi: 10.1371/journal.pone.0153737 (PMC4839612; doi:10.1371/journal.pone.0153737)
Supplement: S1 Appendix — (PDF) [file pone.0153737.s001.pdf]

Appendix A. Canonical Correlation Analysis (CCA) versus linear regression

The purpose of this appendix is to further explain Canonical Correlation Analysis (CCA), the vector-field equivalent of linear regression, and to compare how this differs from scalar linear regression. The goal of CCA is to determine the strength of linear correlation between a set of predictor variables and a set of response variables. We use the specific example of a CCA analysis of the peak hip angle (predictor variable) versus the {HM,VL} vector (response variable).

The result of this CCA is presented below in figure SA.1. The SPM{χ^2} trajectory indicates the magnitude of the canonical correlation between the peak hip angle and the {HM,VL} vector. The maximum canonical correlation occurs at time=49% though the relationship is significant between 43-56% and 78-81% time. The linear relationships between peak hip angle versus HM and peak hip angle versus VL at time=49% are shown in the second column. At this time instance, the relationship between peak hip angle and HM is weak (r=-0.125) but with VL is good (r=0.501). So why is the maximum canonical correlation most significant at this time point? The top right figure plots HM versus VL. The blue line shows a 2D visualisation of the direction of maximum effect on the HM, VL plane. Its true orientation can be described as a 2D plane orientated to maximise the effect in the 3D space of HM, VL and peak hip flexion. When the data are projected onto the direction of maximum effect, the strongest relationship between the {HM,VL} vector is now observed (r=0.523). The direction of maximum effect may change over time to maximise the relationship between the predictor and response variables hence the results of the CCA cannot be directly related to individual component linear regressions unless one vector component is completely unrelated to the predictor variable.

For further details of the calculation process please refer to:

Pataky, T., Robinson, M., Vanrenterghem, J. (2013). Vector field statistical analysis of kinematic and force trajectories. Journal of Biomechanics, 46, 2394-2401. Appendix E.

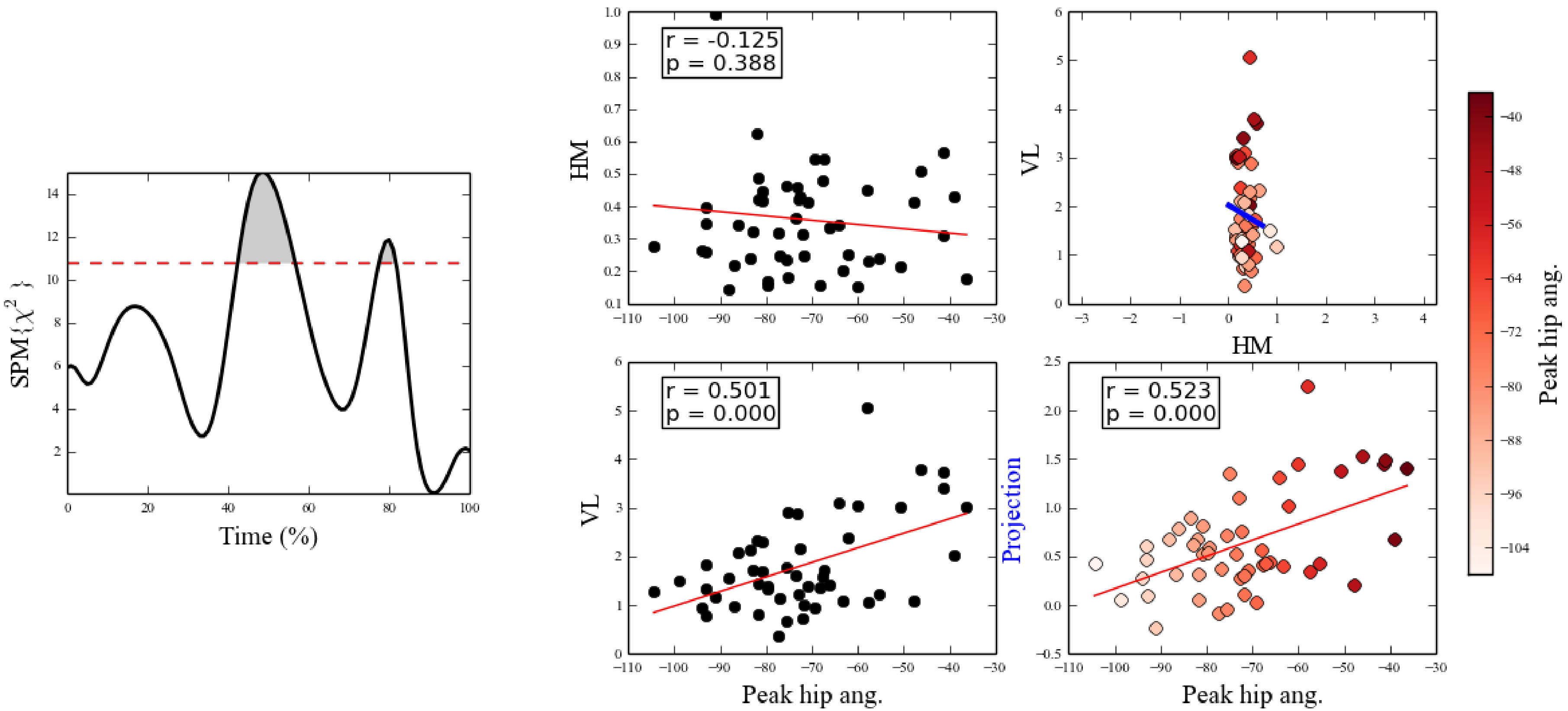

Figure SA.1. Column 1: CCA for peak hip angle (predictor variable) versus the {HM,VL} vector. Column 2: Linear regression of peak hip angle versus HM (top) and VL (bottom). Column 3: (top) HM versus VL with colour indicating the peak hip angle. The blue line represents the direction of maximum effect, (bottom) the peak hip angle projected onto the direction of maximum effect.
